# Transitional Metaplasia of Renal Collecting Ducts in Vesicoureteral Reflux and Chronic Pyelonephritis: An Immunohistochemical Study of a Case and Review of the Literature

**DOI:** 10.30699/IJP.2023.1998769.3087

**Published:** 2023-10-15

**Authors:** Naser Tayyebi Meibodi, Salman Soltani, Farnaz Torabian

**Affiliations:** 1 *Department of Pathology, Cutaneous Leishmaniasis Research Center, Mashhad University of Medical Sciences, Mashhad, Iran*; 2 *Kidney Transplantation Complications Research Center, Mashhad University of Medical Sciences, Mashhad, Iran*; 3 *Department of Pathology, Faculty of Medicine, Mashhad University of Medical Sciences, Mashhad, Iran*

**Keywords:** Chronic renal failure, Kidney collectingducts, Metaplasia, Vesicoureteral reflux

## Abstract

The capability of the urinary tract to undergo metaplastic changes such as squamous, intestinal, glandular, mucinous, or ciliated epithelium in renal pelvis has been previously reported, which hypothetically is due to the mechanical irritation of the transitional epithelium. However, transitional metaplasia is a rare presentation in the collecting ducts. The aim of this paper was to report this type of extremely rare metaplasia and to inform pathologists that they may encounter this kind of metaplasia. A 25-year-old man, a known case of vesicoureteral reflux (VUR), referred to the Imam Reza Hospital; affiliated to the Mashhad University of Medical Sciences, for bilateral nephrectomy. Gross evaluation of bilateral nephrectomy specimens showed atrophic kidneys and dilated pelvicalyceal systems. The light microscopic evaluation showed transitional metaplasia in the background of chronic pyelonephritis, confirmed by GATA3 nuclear immunohistochemical stain. In this study, we presented a rare case of a renal collecting duct with transitional epithelial lining replacing the normal epithelium as a metaplastic change, with the hypothesis that previous medical history including VUR, or hemodialysis could be the trigger for the metaplastic change, which should be confirmed by further studies.

## Introduction

Vesicoureteral reflux (VUR) is a congenital abnormality in children, which may cause recurrent urinary infection. VUR is sub-classified into primary or secondary, based on its etiology. It may be unilateral or bilateral. The incidence of bilateral VUR ranges from 30% to 58% of all children. Furthermore, VUR is one of the remarkable causes of the chronic kidney disease (CKD) in children (1). Metaplastic changes are frequently observed in the histologic microscopic evaluation, which are in relation to the inflammatory stimuli, due to the infections or calculi (2).

 Some papers have reviewed the capability of the urinary tract epithelium to undergo metaplasia from transitional to squamous (3), intestinal, glandular, mucinous, or ciliated epithelium in renal pelvis and is attributed to the mechanical irritation of the urothelium, usually by the calculi (3-12). However, transitional metaplasia of collecting ducts is a rare metaplastic change in collecting ducts in VUR and chronic pyelonephritis. Based on the literature search, there is one letter to editor report that revealed transitional and mucous metaplasia in renal collecting ducts and renal papilla (13). Here, we report a case of transitional metaplasia of the medullary collecting ducts occurring in association with VUR and chronic pyelonephritis.

## Case Presentation

In October 27, 2022, a 25-year-old male with a history of urinary tract infection since 16 years ago, referred to the Urology Clinic. He was admitted to the Imam Reza Hospital, affiliated to the Mashhad University of Medical Sciences for bilateral nephrectomy. He was the known case of bilateral VUR, grade 4 for 16 years ago and neurogenic bladder along with the following chronic renal failure (CRF) since 5 years ago without evidence of nephrolithiasis. He had a history of being surgically treated for the neurogenic bladder on a number of occasions. He has been on hemodialysis since 5 years ago along with medical intervention. Voiding cystourethrogram (*VCUG*) showed VUR, grade 4. Investigations reveled urea: 71 mg/dL, creatinine: 7 mg/dL, sodium: 133 mEq/L, potassium: 6 mEq/L, Hb: 9.4 g/dL, and total leukocyte count 7000/mm^3^. He is now waiting for the kidney transplantation. 

**Fig. 1:**
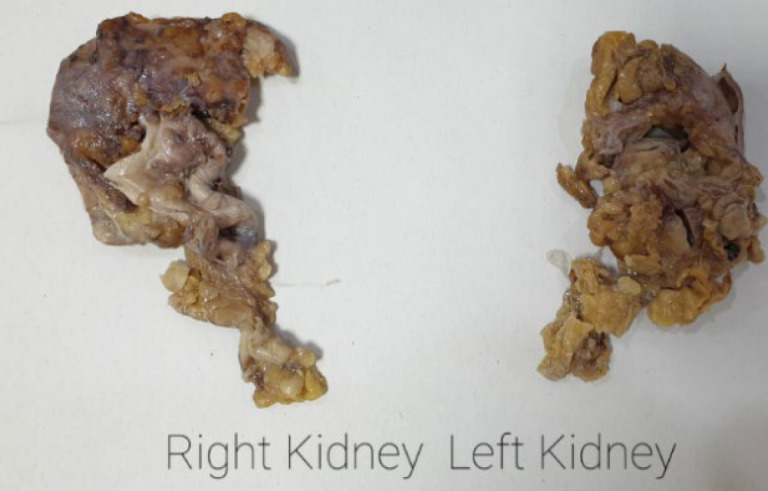
Gross appearance of atrophic right and left kidneys

On gross examination, each right and left nephrectomy specimen weighted 50 gram and measured 8×3×2 cm and 7×2×2 cm, respectively; consistent with atrophic appearance (Figure 1). On cut surface, grossly dilated right pelvicalyceal system was present with narrow rim of renal parenchyma.

**Fig. 2:**
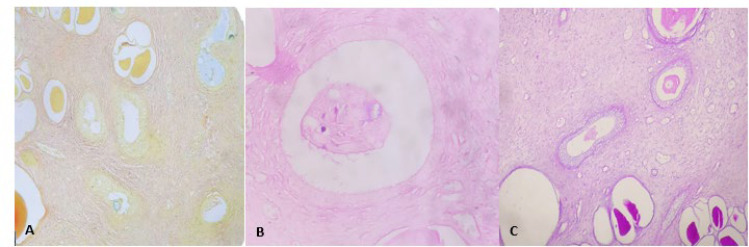
**A**. Negative for colloidal iron stain in transitional metaplasia of collecting ducts (X400). **B**. Negative Alcian blue stain in collecting ducts with transitional metaplasia, (X400). **C**. Negative PAS staining in the apical cytoplasm of metaplastic transitional ductal epithelium and PAS positive material in the lumen of some collecting ducts, (X400).

**Fig. 3. A, B:**
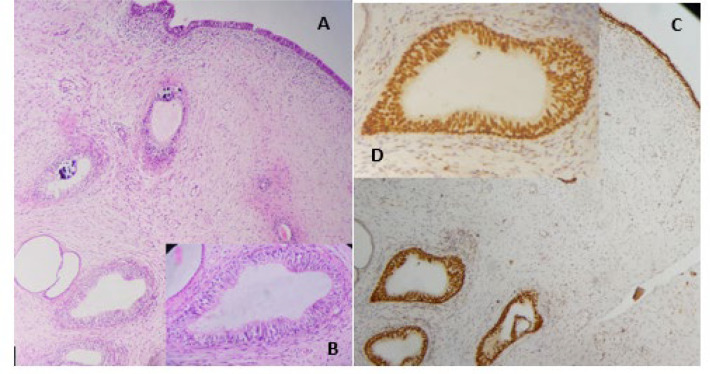
Sections show renal papilla collecting ducts with transitional metaplasia (H&E stain, 100X, 400X). **C, D**. GATA3 nuclear immunohistochemical staining in transitional metaplasia of the collecting ducts (IHC stain 100X, 400X).

Microscopic examination on hematoxylin and eosin (H&E) stained sections of the renal parenchyma revealed the characteristic changes of chronic pyelonephritis composed of interstitial fibrosis, inflammation, intimal fibrosis, and thyroidization along with collecting ducts lined by metaplastic transitional epithelium. In Periodic acid Schiff (PAS) staining, negative staining in the apical cytoplasm of metaplastic transitional ductal epithelium and PAS positive material in the lumen of some collecting ducts were present (Figure 2). Immunohistochemical staining was performed to evaluate the transitional metaplasia using GATA3 nuclear staining (Figure 3). Colloidal iron and Alcian blue stains in metaplastic epithelium were negative (Figure 2).

## Discussion

In this study, we presented an extremely rare case with renal collecting duct showing transitional epithelial metaplasia. Metaplastic changes of the renal pelvis include squamous, intestinal, and ciliated metaplasia that have been previously reported (2, 3, 5). A study done by Mathur *et al*. identified mucinous glandular metaplasia of renal pelvis in the setting of recurrent renal calculi, chronic pyelonephritis, and pyonephrosis. They also specified type of mucin by the special stains in metaplastic epithelium. The foci stained with Alcian blue PAS (AB-PAS), showed small intestinal metaplasia, with both sialomucins and neutral mucin. The definitive mechanism of glandular metaplasia is not clear. It has been hypothesized that embryonal cloaca and intestine are both of endodermal origin (14).

In a recent paper, Rullo *et al.* illustrated ciliated pseudostratified columnar cells in the renal calyx mucosa adjacent to the invasive urothelial carcinoma presented in 82-year-old man suffered from nephrolithiasis. The immunohistochemical evaluation revealed positive keratin 7 and keratin 8/18 and negative keratin 20 results. The special staining including Alcian blue was positive in some vacuoles of apical cytoplasm of the same cells, while PAS was negative in the same cells. The GATA3 immunohistochemical stain was positive only in the basal layer of the epithelium but not in the ciliated cells (2), in contrast to our study with full thickness staining of metaplastic epithelium. Urolithiasis seems to be responsible for this unusual cell change, hypothetically. All hypotheses, need to be confirmed by further studies.

Only one report in the literature has documented the finding of transitional and mucous metaplastic changes in the renal collecting ducts and renal papilla in a patient with cellular rejection of the kidney with prominent diffuse interstitial mononuclear cell infiltration in cortex and medulla (13).

Metaplasia is characterized as a protective response due to exposure to the chronic etiologies. Also, due to the narrower lumen of minor calyces, obstruction occurs more easily, resulting in some responses such as keratinizing squamous metaplasia in renal collecting ducts and proximal structures of the kidney (3). Given the case presented here, a permanent injury should be considered as a possible etiology. According to the adult mouse unilateral obstruction (UUO) model explained by Girshovich *et al.*, an increased intrapelvic pressure may alter intra-renal epithelium into a bladder-like urothelium composed of the multilayered epithelium with strong expression of uroplakins Ib, II, and III (15). Thus, chronic VUR may be responsible for this rare metaplastic change. Another possibility that should be considered, is the presence of metaplasia due to hemodialysis. In the same direction with this hypothesis, some authors have reported squamous metaplasia in tubules in the setting of the end-stage kidney dialysis (13). In our case, some collecting ducts in medulla were lined by a transitional metaplastic epithelium. Further studies are needed to evaluate the main reason for this rare metaplastic change. The aim of this report was to point this type of extremely rare metaplasia and to inform pathologists about the possibility of such type of metaplasia in their practice.

## Funding

The authors received no financial support for this research.

## Conflict of Interest

The authors declared no conflict of interest.
